# Efficient Double Fragmentation ChIP-seq Provides Nucleotide Resolution Protein-DNA Binding Profiles

**DOI:** 10.1371/journal.pone.0015092

**Published:** 2010-11-30

**Authors:** Michal Mokry, Pantelis Hatzis, Ewart de Bruijn, Jan Koster, Rogier Versteeg, Jurian Schuijers, Marc van de Wetering, Victor Guryev, Hans Clevers, Edwin Cuppen

**Affiliations:** 1 Cancer Genomics Center, Hubrecht Institute and University Medical Center Utrecht, Utrecht, The Netherlands; 2 Department of Human Genetics, Academic Medical Center, University of Amsterdam, Amsterdam, The Netherlands; 3 Department of Medical Genetics, University Medical Center Utrecht, Utrecht, The Netherlands; University College London, United Kingdom

## Abstract

Immunoprecipitated crosslinked protein-DNA fragments typically range in size from several hundred to several thousand base pairs, with a significant part of chromatin being much longer than the optimal length for next-generation sequencing (NGS) procedures. Because these larger fragments may be non-random and represent relevant biology that may otherwise be missed, but also because they represent a significant fraction of the immunoprecipitated material, we designed a double-fragmentation ChIP-seq procedure. After conventional crosslinking and immunoprecipitation, chromatin is de-crosslinked and sheared a second time to concentrate fragments in the optimal size range for NGS. Besides the benefits of increased chromatin yields, the procedure also eliminates a laborious size-selection step. We show that the double-fragmentation ChIP-seq approach allows for the generation of biologically relevant genome-wide protein-DNA binding profiles from sub-nanogram amounts of TCF7L2/TCF4, TBP and H3K4me3 immunoprecipitated material. Although optimized for the AB/SOLiD platform, the same approach may be applied to other platforms.

## Introduction

**Figure 1:**
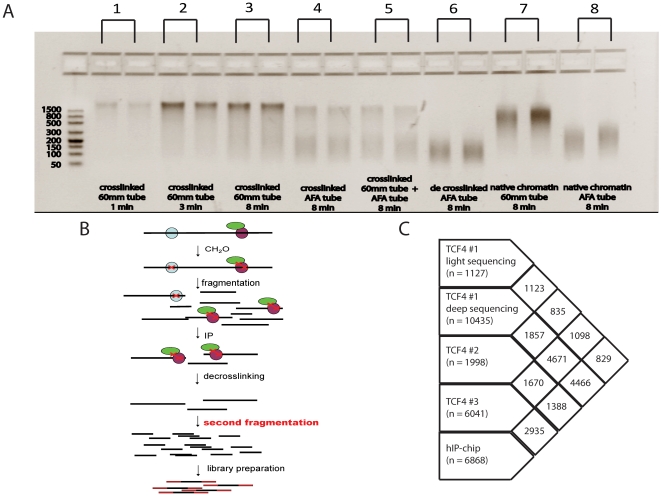
Double fragmentation ChIP-seq approach. A) Comparison of different shearing methods on crosslinked, de-crosslinked and native chromatin. Samples 1–3 represent crosslinked chromatin sheared at the same power intensity with increasing shearing times in 60 mm tubes, sample 4 is crosslinked chromatin sheared using AFA tubes (Covaris), sample 5 is crosslinked chromatin sheared using 60 mm tubes and subsequently sheared in AFA tubes, sample 6 is crosslinked chromatin sheared in 60 mm tubes, de-crosslinked and subsequently sheared in AFA tubes, samples 7 and 8 are samples of native chromatin sheared using 60 mm tubes and AFA tubes, respectively. Extensive shearing of crosslinked chromatin (e.g. sample 5) still leaves a significant proportion of chromatin fragments outside the optimal range for next-generation sequencing. However, this fraction can be sheared to smaller fragments after de-crosslinking (sample 6), but not without de-crosslinking (sample 5). B) Schematic overview of the double fragmentation ChIP-seq procedure. After normal immunoprecipitation, DNA is de-crosslinked, purified and additionally sheared to concentrate all fragments in the size range that is optimal for short tag sequencers like AB/SOLiD (100–300 nt) or Illumina/Solexa (400–600 nt). C) Overlap between TCF4 ChIP-chip and ChIP-seq data. Peak sets from libraries prepared with the double shearing approach show a larger overlap with the ChIP-chip peak data.

ChIP-seq has become the method of choice for studying functional DNA-protein interactions on a genome-wide scale. The method is based on the co-immunoprecipitation of DNA binding proteins with formaldehyde cross-linked DNA, followed by deep-sequencing of the immunoprecipitated chromatin fragments. This allows for the genome-wide identification of binding sites with high accuracy [1], [2], [3], [4], [5]. Typical immunoprecipitated DNA fragments range in size from several hundred to several thousand base pairs. As a result, a significant part of the chromatin is not in the optimal size range for direct application to next-generation sequencing (Fig. 1A). In current ChIP-seq approaches immunoprecipitated DNA fragments within the optimal sequencing range (100–200 base pairs for AB/SOLiD or 300–500 for Solexa/Illumina) are typically size-selected by gel-excision and converted into sequencing libraries followed by next-generation sequencing. However, this approach discards large amounts of specifically immunoprecipitated material in the larger size range, thereby increasing the demands on the amount of starting material. Furthermore, it could be possible that the observed size distribution is not random and reflects specific biology [6].

To address these limitations we applied a strategy with a first gentle shearing step before immunoprecipitation and a second more intensive shearing of purified de-crosslinked DNA after immunoprecipitation to additionally fragment all material into small fragments suitable for next-generation sequencing. We have optimized our protocols for sequencing on the SOLiD/AB platform, with an optimal fragment size of 100–200 bp, but this size range can be adapted at will. Furthermore, we show that the size range after the second fragmentation step is so narrow that it is possible to skip a laborious size selection in the library preparation procedure.

To demonstrate general utility, we performed ChIP-seq according to this protocol for well-characterized factors such as TBP, H3K4me3, and TCF7L2/TCF4, one of the members of the Tcf/Lef family of Wnt pathway effectors [7], [8], [9]. Consensus TCF4 binding sites have been biochemically determined [9] and genome-wide binding profiles for TCF4 in colon cancer cells have been determined previously by ChIP-on-chip experiments [10]. The results obtained here are in strong concordance with these previous results.

## Results and Discussion

### Double fragmentation ChIP method

When shearing cross-linked DNA, a significant part of cross-linked chromatin remains too long for direct processing for next-generation sequencing, irrespectively of the fragmentation procedure (Fig. 1A). As a consequence, a major part of the immunoprecipitated chromatin would be discarded after size selection and increases the required amount of starting material. Therefore, we introduced a double-fragmentation method for processing ChIP–seq samples with a second intensive shearing of decrosslinked immunoprecipitated chromatin into fragment lengths that are optimal for next-generation sequencing platforms (Fig. 1B). This approach provides the possibility to use less starting material compared to conventional methods, as virtually all DNA is concentrated in the desired optimal size range for downstream processing. Although the size range of the second shearing step can be adjusted to any size range between 100 and 500 bp, we have focused on optimization for the AB/SOLiD platform. To demonstrate this, we split one of the TCF4-immunoprecipitated samples into 2 equal parts and prepared 2 independent libraries that were size-selected for fragments that are optimal for SOLiD emulsion PCR and sequencing, one with and one without second fragmentation. The library produced with the double-fragmentation method resulted in much more unique reads (1.93 M unique reads from 3.4 M mapped reads) as compared to the sample processed without secondary fragmentation (0.89 M unique reads from 3.4 M mapped reads), for this purpose when two or more reads have the same starting position on the same strand they were counted as single unique read. Although in both cases it was possible to prepare a sequencing library, the library prepared by the double shearing method is much richer in unique fragments compared to the library prepared without second shearing, indicating that amounts of chromatin (700 pg) were limiting factor in the later case. In the double-fragmentation experiments presented here, we successfully used amounts as low as 0.7 ng of immunoprecipitated DNA for sample preparation, which is much less than the 10 ng that is recommended as the lowest amount in standard ChIP-seq protocols [11]. These results indicate that the double-fragmentation ChIP-seq protocol may be well suited for challenging experiments, for example with lower affinity antibodies or in cases where only limited amounts of source material is available. Furthermore, the larger chromatin fragments may be non-random and due to specific biology [6], e.g. packed in large DNA-protein complexes. In support of this, a second rigorous shearing of crosslinked DNA could not fragment all chromatin in small fragments (Fig. 1A, sample 5), whereas the same treatment on de-crosslinked DNA results in subfractionation of almost all chromatin in the desired size range (Fig. 1A, sample 6).

### Comparison of double fragmentation ChIP-seq with ChIP-chip data

Three independent TCF4 ChIP samples obtained from the human colon cancer cell line Ls174T were prepared at different time points as described previously [10]. These samples were converted into sequencing libraries using the double fragmentation approach and analyzed by AB/SOLiD sequencing (Table 1) (raw sequencing files, alignment files and called peaks have been submitted into GEO database with accession number: GSE18481). In addition, sample 1 was sequenced twice at variable depth. In the case of sample 3, DNaseI was used for second fragmentation. We identified 948 to 10,435 binding regions with the number of identified peaks strongly depending on sequencing depth and false discovery rate settings (Table 1).

**Table 1:** TCF4 ChIP libraries overview.

Sample	Fragmentation method	Uniquely mapped reads (millions)	Number of peaks(0.1 FDR)	Number of peaks(0.01 FDR)	Peaks (0.1 FDR) overlapping with 6,868 high confidence ChIP-chip peaks [10]	Peaks (0.1FDR) overlapping with 11,912 ChIP-chip peaks [10]
TCF4 #1(1^st^run)	sonication	1.2	1,127	948	829	851
TCF4 #1(2^nd^run)	sonication	16.9	10,435	6,638	4,466	5,302
TCF4 #2	sonication	4.5	1,998	1,493	1,388	1,417
TCF4 #3	DNaseI	7.4	6,041	4,135	2,935	3,217
TBP	sonication	26.0	8,734	7,303	NA	NA
H3K4me3	sonication	9.2	15,671	15,411	NA	NA

*NA – not applicable*.

The results from all libraries showed a strong overlap with each other and with a previously published set of peaks that were obtained by ChIP-on-chip [10] (Fig. 1C). Since virtually all of the large peaks identified from libraries prepared by the double fragmentation method do overlap nicely with the ChIP-chip dataset, we can conclude that no major artifacts are introduced by the double fragmentation procedure. Even from the first test sequencing run of Sample 1, where only 1.2 millions of uniquely mapped sequencing reads were generated, 1,127 binding regions were called, out of which 829 (73.5%) mapped to a previously published set of 6,868 high-confidence peaks as determined by ChIP-on-chip [10]. However, data from the other experiments illustrate that determination of the complete genome-wide set of TCF4 binding sites is complex and that the number of peaks strongly depends on sequencing depth and enrichment efficiency of the ChIP. Our results also suggest that many weaker TCF4 binding sites exist, which are likely missed by ChIP-on-chip or lower-depth ChIP-seq [12]. However, it remains to be demonstrated if these ‘weaker’ peaks are of biologic relevance.

### Versatility and simplification of the procedure

To demonstrate general utility of the described method we processed chromatin immunoprecipitation samples of TATA-binding protein (TBP) and H3 lysine 4 tri-methylation histone mark (H3K4me3) in the same way (Table 1). Since virtually all chromatin fragments after the second fragmentation were in the range that is suitable for SOLiD/AB sequencer, the size selection step during library preparation was omitted for these samples. Peaks called from TBP (n = 8,734) and H3K4me3 (n = 15,671) ChIP libraries were predominantly found within 5 kb from transcription start sites of protein coding genes (65,0% in case of TBP and 79,7% in case of H3K4me3) with only a smaller subset of peaks mapping elsewhere (mostly close to non-coding RNAs and/or possibly non-annotated transcripts), which is in line with published results [13], [14].

### Analysis of peak substructure

**Figure 2:**
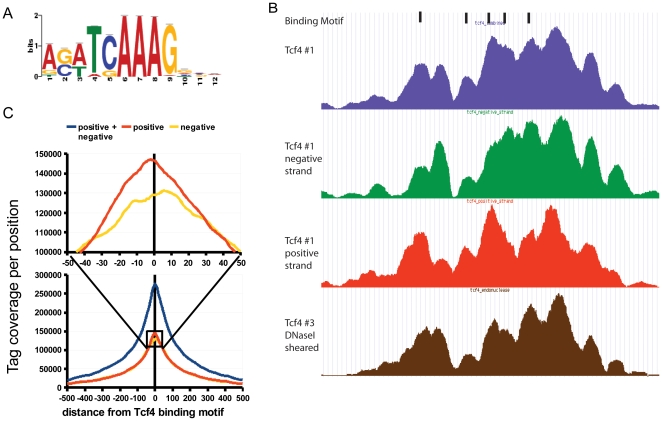
Substructure of binding regions. A) Consensus binding motif sequence logo as identified by Cisgenome from the ChIP-seq data. B) Comparison of Tcf4 binding regions reconstructed from the reads mapped to both strands (blue), the negative strand (green), the positive strand (red), all sub-fragmented using sonication, and reads derived from a library sub-fragmented with DNaseI (brown). The shape and structure of the binding region is highly similar for both strands and does not depend on the fragmentation method used. C) Distribution of sequencing tags from positive and negative strands around the consensus TCF4 binding motif. In contrast to existing protocols without additional fragmentation the maxima of the peaks called separately from the positive and the negative strand overlap with only minor shifting.

The double fragmentation ChIP-seq approach was found to provide a very high resolution with clear substructures in the larger peaks (Fig. 2B). As the second fragmentation step using sonication could potentially introduce a shearing bias, we used a different method, employing partial DNase I digestion to exclude an artificial origin of the observed substructure. A near identical peak substructure was identified excluding fragmentation bias as the origin of the observed patterns and indicating that the observed substructure has a biological rather than technical origin (Fig. 2B), however effects of PCR amplification and/or variation in context-dependent sequencing efficiency cannot be excluded as factors that contribute to the observed patterns.

Indeed, we found that the substructure pattern readily allows for the identification of TCF4 binding positions (Fig. 2). Individual binding events of TCF4 as determined by the presence of the canonical binding motif (Fig. 2A) were found in the tops of the peaks without the need for any computational deconvolution. In line with this and in contrast to existing protocols [4], [5] virtually the same peak pattern is obtained when calling peaks separately from the negative and positive strands (Fig. 2B). In addition, the distribution of sequencing reads around the TCF4 binding motifs of peaks with only one binding motif shows that the motif is present in the center of the peak with only a small shift of about 10 bp between the tops of the positive and negative strand peaks (Fig. 2C). In contrast, for most commonly used approaches, only the ends of immunoprecipitation products are sequenced, resulting in sequencing coverage peaks flanking the real binding site. Typically, the tops of the + and - strand peaks are separated by up to 200 nt [5].

### Biological relevance of binding sites found by double fragmentation method

**Figure 3:**
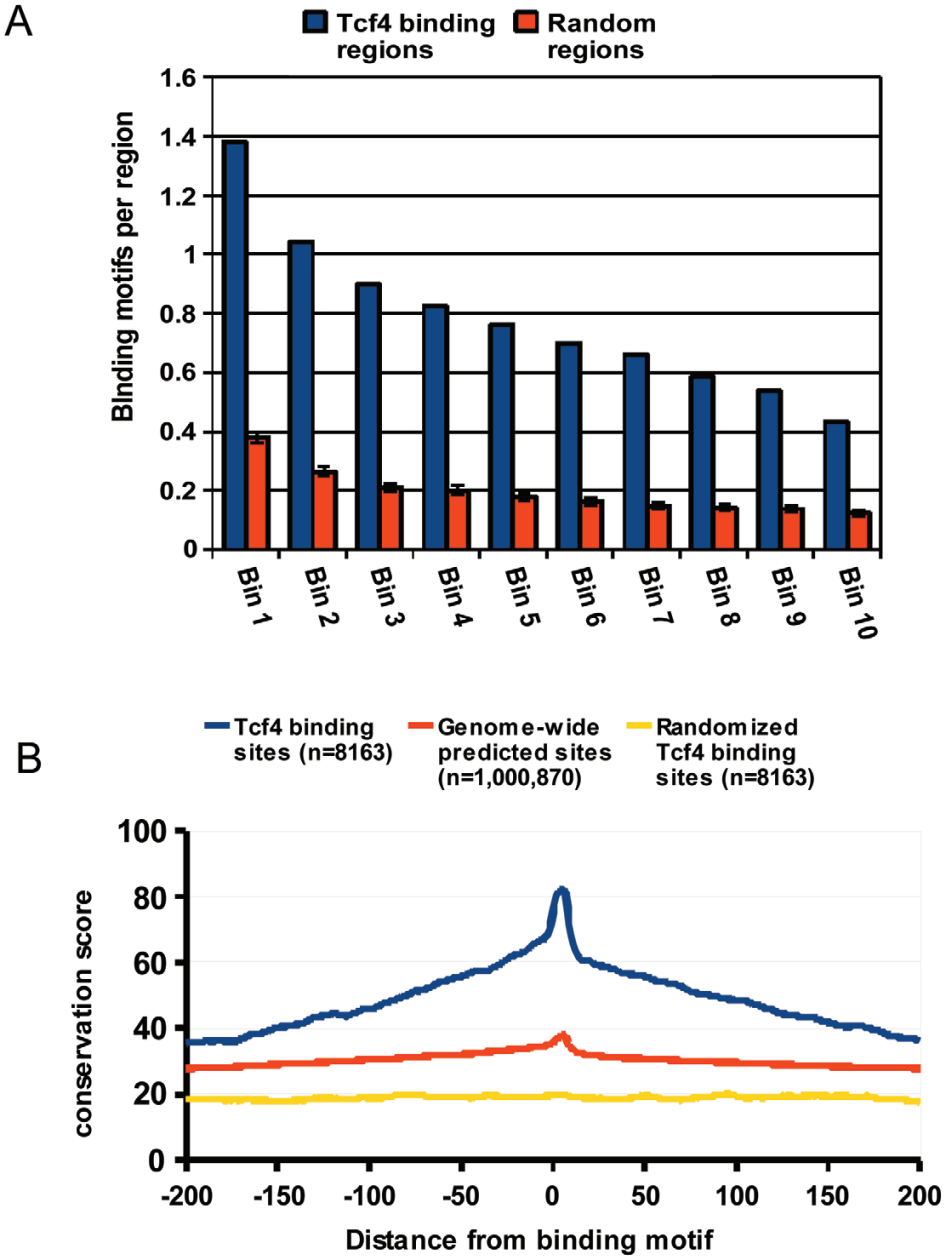
Characterization of TCF4 binding peaks. A) Number of peaks with at least one TCF4 binding motif in relation to the protein-DNA interaction strength. Peaks containing more reads (lower bin numbers) more often harbor a TCF4 binding motif compared to weaker ones. B) Conservation profile of experimentally identified TCF4 binding regions as well as all genomic regions containing the TCF4 consensus binding motif as compared to random regions. Experimentally identified binding regions were found to be more conserved than computationally predicted sites.

Cisgenome [15] was used to identify overrepresented consensus motifs within the immunoprecipitated regions. The most common motif discovered (Fig. 2A) was nearly identical to the consensus TCF4 binding motif that has been described previously [9], [10]. From 10,435 binding sites, 55.5% contain at least one TCF4 binding motif. Larger peaks, as defined by the number of reads in the peak, more often have TCF4 binding motifs compared to weaker ones (Fig. 3A). In addition, 2,369 (22.7%) peaks contain two or more TCF4 binding motifs. This observation is in concordance with accepted models where the presence of several binding motifs close to each other increases the probability that the transcription factor spends more time bound to a particular region [16]. In addition, by analyzing binding motifs found in TCF4 binding regions we were able to identify known and potentially novel transcription factors that interact with TCF4 (Figure S1 and Figure S2).

To explore the evolutionary conservation of the observed TCF4 binding sites we used the phastCons [17] scores for each position in the binding regions. Both 200-nt long neighboring flanking sequences and 12-nt long TCF4 binding motifs were more conserved compared to random genomic locations, where the conservation score of the TCF4 binding motif was on average higher than neighboring flanking regions (Fig. 3B), indicating selective pressure on these motifs and pointing to functional relevance.

In contrast to ChIP-chip, ChIP-seq has a very high dynamic range and allows for semi-quantitative estimation of DNA-protein interaction strength (as a function of the number of sequencing reads which mapped to the binding region) [5]. We divided peaks in bins according to the interaction strength and studied their characteristics. Interestingly, weaker peaks were found enriched towards transcription start sites (TSS). As these peaks were also found to more often lack a consensus TCF4 site, it is likely that these regions represent co-immunoprecipitated chromatin that interacts indirectly via DNA-looping with TCF4-containing protein-DNA complexes, similarly as described previously [18] (Figure S3 and Figure S4).

### Correlation of sites found by double fragmentation method with differential gene expression

**Figure 4:**
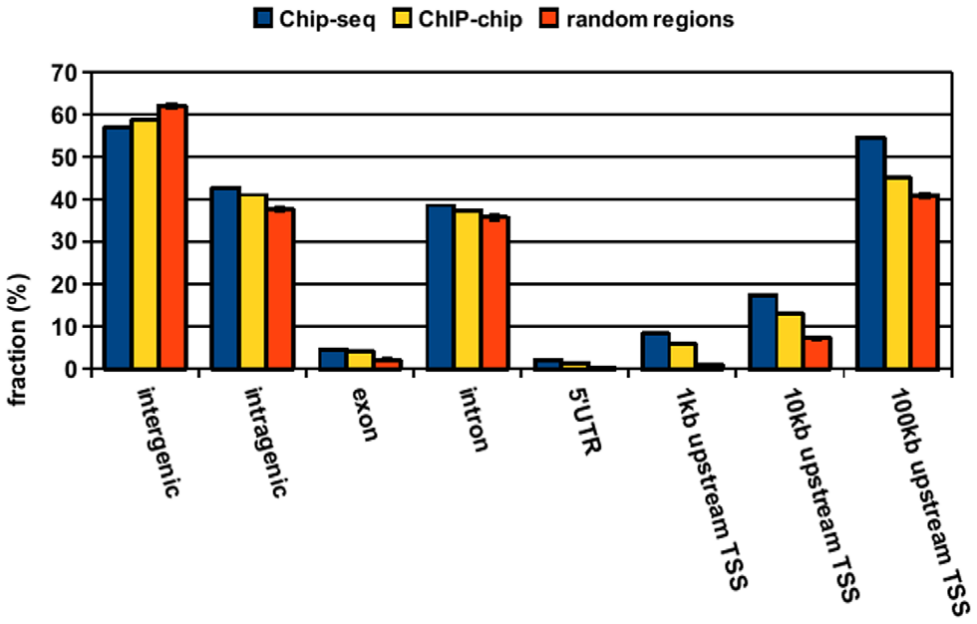
Distribution of TCF4 binding peaks. The distribution of the TCF4 ChIP-seq peaks was analyzed with respect to the closest gene and compared to the distribution of random regions. Genome-wide distribution of ChIP-seq peaks is similar to those identified previously by ChIP-chip with peaks predominantly located far from annotated transcription start sites. This is in line with the established role of TCF4 as a transcriptional enhancer. Error bars for random regions represent standard deviation of 100 randomized datasets.

**Figure 5:**
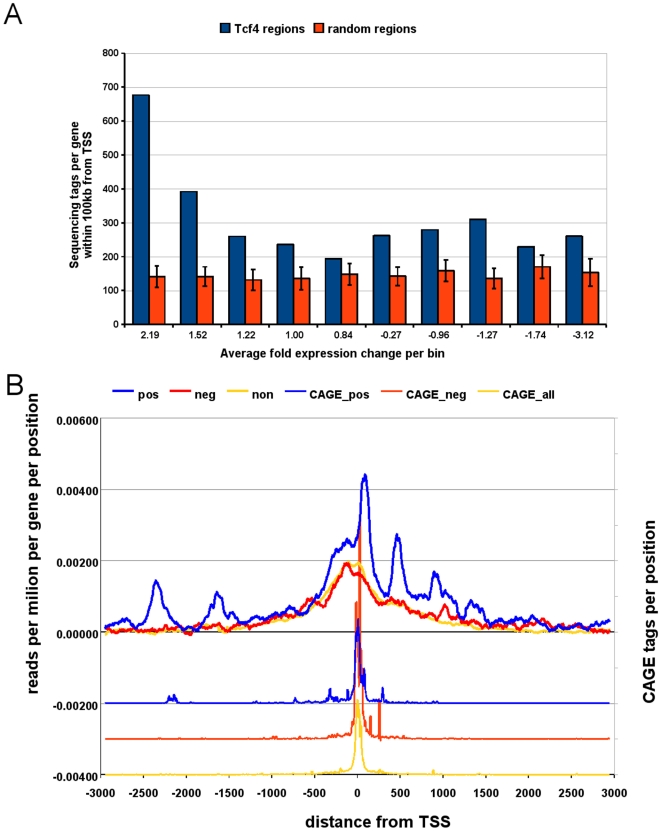
Distribution of binding regions with respect to Wnt regulated genes. A) Gene expression rank analysis. Genes positively regulated by Wnt contain more peak forming sequencing tags within 100 kb from their transcription start sites B) Enrichment pattern of sequencing reads around TSS of up-, down-, and non-regulated genes. The observed pattern with additional maxima downstream and upstream of TSS could potentially be explained partially by the presence of alternative or non-annotated TSS, which is actually supported by the presence of CAGE tags in those regions.

The genome-wide distribution of TCF4 binding sites with respect to TSS of the nearest gene shows a similar distribution as previously reported [10] (Fig. 4). A substantial proportion of the peaks is located more than 10 kb from the closest TSS, supporting the model of long range regulation of gene expression by TCF4. This pattern is also in line with the distribution of other sequence-specific transcription factors such as estrogen receptor [19], STAT1 [2], Foxa2 [3] and p53 [20]. To unravel functional TCF4 regulatory gene expression modules connected to Wnt signaling, we used microarray-based gene expression data from modified Ls174T colorectal cancer cell lines (Table S1) (microarray data have been submitted to GEO database with accession number: GSE18560). Inducible overexpression of a dominant negative form of TCF4 or siRNA against β-catenin, as described previously [21], [22] was used to conditionally turn Wnt signaling off. Genes were ranked according to induced expression changes after abrogation of Wnt signaling and analyzed for the presence of TCF4 immunoprecipitated binding regions. Genes with decreased expression after ablation of the Wnt pathway and thus positively regulated by Wnt and TCF4 had more peak-forming sequencing tags within 100 kb from their transcription start site compared to genes with less prominent changes in expression or down-regulated genes (Fig. 5A). Although sequencing reads were found to be enriched over the TSS of all 3 groups of genes – up-regulated, down-regulated and non-differentially regulated - enrichment was most prominent for actively up-regulated genes and comparable for negatively and non-regulated genes (Fig. 5B).

### Concluding remarks

Starting from sub-nanogram amounts of immunoprecipitated chromatin we show that the double-fragmentation ChIP-seq protocol allows for the accurate determination of genome-wide binding patterns at high resolution. We show that the method is highly reproducible and versatile and can serve as an alternative for current ChIP-seq protocols especially when limited amounts of immunoprecipitated material are available. Although optimized for the AB/SOLiD platform, shearing settings could be adjusted for the optimal size range for other platforms (e.g. Illumina/Solexa) as well. Most importantly, the biological relevance of the resulting datasets was firmly demonstrated by in-depth analysis of TCF4 binding regions.

## Materials and Methods

### Cells

Ls174T human colon cancer cells carrying an activating point mutation in beta-catenin were used throughout this study. Ls174T-L8 cells carry a doxycyclin-inducible dominant-negative TCF4 transgene; Ls174T-pTER-β-catenin cells carry a doxycyclin-inducible shRNA against β-catenin; they allow for complete and specific blocking of the constitutively active Wnt pathway [21], [22].

### ChIP

Chromatin immunoprecipitation was performed as described previously [10]. In brief, Ls174T cells were cross-linked with 1% formaldehyde for 20 min at room temperature. The reaction was quenched with glycine at a final concentration of 125 mM. The cells were successively washed with phosphate-buffered saline, buffer B (0.25% Triton-X 100, 10 mM EDTA, 0.5 mM EGTA, 20 mM HEPES [pH 7.6]) and buffer C (0.15 M NaCl, 1 mM EDTA, 0.5 mM EGTA, 20 mM HEPES [pH 7.6]) at 4°C for 10 min each. The cells were then resuspended in ChIP incubation buffer (0.3% sodium dodecyl sulfate [SDS], 1% Triton-X 100, 0.15 M NaCl, 1 mM EDTA, 0.5 mM EGTA, 20 mM HEPES [pH 7.6]) and sheared using a Bioruptor sonicator (Cosmo Bio Co., Ltd.) with six pulses of 30 s each at the maximum setting (Library 1 and 2) or using a Covaris S2 (Covaris) for 8 minutes with the following settings: duty cycle: max, intensity: max, cycles/burst: max (Library 3, TBP, H3K4me3). Both approaches produced similar DNA fragment size range distributions. The sonicated chromatin was incubated for 12 h at 4°C with the appropriate antibody (polyclonal anti-TCF4 antibody, sc-8631; Santa Cruz Biotechnology, Inc.; polyclonal Anti-trimethyl-Histone H3 (Lys4), 07-473, Millipore; TATA binding protein TBP antibody [1TB18] - ChIP Grade, ab12089, Abcam) at 1 µg of antibody per 10^6^ cells with 150 µl of protein G beads (Upstate). The beads were successively washed 2 times with buffer 1 (0.1% SDS, 0.1% deoxycholate, 1% Triton-X 100, 0.15 M NaCl, 1 mM EDTA, 0.5 mM EGTA, 20 mM HEPES [pH 7.6]), one time with buffer 2 (0.1% SDS, 0.1% sodium deoxycholate, 1% Triton-X 100, 0.5 M NaCl, 1 mM EDTA, 0.5 mM EGTA, 20 mM HEPES [pH 7.6]), one time with buffer 3 (0.25 M LiCl, 0.5% sodium deoxycholate, 0.5% NP-40, 1 mM EDTA, 0.5 mM EGTA, 20 mM HEPES [pH 7.6]), and two times with buffer 4 (1 mM EDTA, 0.5 mM EGTA, 20 mM HEPES [pH 7.6]) for 5 min each at 4°C. The precipitated chromatin was eluted by incubation of the beads with elution buffer (1% SDS, 0.1 M NaHCO_3_) at room temperature for 20 min, the eluted fraction was reconstituted to 0.3% SDS with ChIP incubation buffer and the immunoprecipitation repeated with half the amount of antibody. After washing and elution, the immunoprecipitated chromatin was de-cross-linked by incubation at 65°C for 5 h in the presence of 200 mM NaCl, extracted with phenol-chloroform, and ethanol precipitated. Measurement of chromatin concentration was done by high-sensitivity Qubit quantitation (Invitrogen).

### Sequencing library preparation

Immunoprecipitated chromatin was dissolved in 100 µl of 10 mM Tris pH 8.0 buffer and sheared for a second time for 6 minutes using the Covaris sonicator (6×16 mm AFA fiber Tube, duty cycle: 20%, intensity: 5, cycles/burst: 200, frequency sweeping) to obtain suitable shorter fragments (75–125 bp). To exclude a shearing bias as a possible source of the observe binding site substructure, half of TCF4 Sample 3 was processed with partial digestion using DNaseI as an alternative to shorten the fragments for one control ChIP-seq library and one input library. The second half of the sample was processed without second fragmentation. For DNaseI treatment, chromatin was resuspended in 45 µl of freshly prepared reaction buffer (10 mM MnCl_2_, 0.1 mM CaCl_2_, 10 mM Tris, pH 7.5) and digested with 0.5 mU of DNaseI for 5 minutes at room temperature. The reaction was stopped by adding EDTA to a final concentration of 50 mM. Chromatin was immediately extracted using phenol/chloroform and precipitated. After fragmentation, the fragments were blunt-ended and phosophorylated at the 5′ end using the End-it Kit (Epicentre) according to the manufacturer's instructions. Ligation of double stranded adapters compatible with SOLiD sequencing was performed using Quick ligation kit (New England Biolabs) with 750 nM P1 and P2 double-stranded adaptors (Applied Biosystems), 11.7 µl of 2× Quick ligation buffer, 1 µl Quick Ligase in a total volume of 23.4 µl. Samples were purified using Ampure beads (Agencourt) and separated on a native 6% polyacrylamide gel. Fragments ranging from 140 to 190 bp were excised; the gel piece containing the selected DNA fragments was shredded and dispersed into 400 µl of Platinum PCR Supermix with 750 nM of each P1 and P2 PCR primer, 2.5 U of Pfu (Stratagene) and 5 U Taq (Bioline). In case of TBP and H3K4me3 samples, the acrylamide gel-based size selection step was skipped and the adapter-ligated library was directly further processed by PCR. Prior to ligation-mediated PCR the sample was incubated at 72°C for 20 minutes in PCR mix to let the DNA diffuse out of the gel and to perform nick translation on non ligated 3′-ends of DNA fragments. After 17 cycles of amplification the library was purified using Ampure beads and was quality checked on 2100 Bioanalyzer (Agilent) for the absence of possible adapter dimers and heterodimers. Concentration of double-stranded DNA in the final sample was determined by Qubit fluorometer (Invitrogen).

### SOLiD sequencing

To achieve clonal amplification of library fragments on the surface of sequencing beads, emulsion PCR (ePCR) was performed according to the manufacturer's instructions (Applied Biosystems). 600 pg of double stranded library DNA was added to 5.6 ml of PCR mix containing 1× PCR Gold Buffer (Applied Biosystems), 3000 U AmpliTaq Gold, 20 nM ePCR primer 1, 3 µM of ePCR primer 2, 3.5 mM of each deoxynucleotide, 25 mM MgCl_2_ and 1.6 billion SOLiD sequencing beads (Applied Biosystems). PCR mix was added to SOLiD ePCR Tube containing 9 ml of oil phase and emulsified using ULTRA-TURRAX Tube Drive (IKA). Emulsion was dispensed into 96-well plate and cycled for 60 cycles. After amplification emulsion was broken with butanol, beads were enriched for template positive beads, 3′-end extended and covalently attached onto sequencing slides. Four physically separated samples were deposited on one sequencing slide and sequenced using standard settings on the SOLiD system version 2 to produce 35 nucleotide long reads. TBP and H3K4me3 libraries were sequenced using AB/Solid version 3 to produce 50 bp long reads.

### Mapping of sequencing data

Sequencing reads were quality trimmed by clipping at 3 consecutive nucleotides with quality score less than 10. Reads shorter than 18 nucleotides were discarded and the remaining reads were mapped against the human reference genome (hg18 assembly, NCBI build 36) using SHRiMP package [23] with default settings, which allows mapping in SOLiD color space corresponding to dinucleotide encoding of the sequenced DNA. For analysis we used only uniquely mapped reads, which were defined as reads having at least two additional mismatches in the second best hit compared to the best hit. TBP and H3K4me3 libraries were mapped against the reference genome (hg18 assembly, NCBI build 36) using the Maq package [24], which allows mapping in SOLiD color space corresponding to dinucleotide encoding of the sequenced DNA with following settings: -n 3, -e 150. Reads with mapping quality zero were discarded.

### Peak identification

The Cisgenome software package [15] was used for the identification of binding peaks from the ChIP-seq data. Two-sample analysis mode was used to compare samples with control input data. The parameters for peak discovery were set as follows: window size 100, step size 25, maximum gap 200, active single strand filtering, minimum peak with 225 and minimum reads per window was set to obtain <0.1 or 0.01 false positive rate (FDR). Peaks called with 0.1 FDR from the second sequencing round of TCF4 Library 1 (n = 10,435) were used as a final set for subsequent genome-wide analyses.

### Characterization of TCF4 binding regions

The analysis of sequencing reads and TCF4 binding regions with respect to gene structure and distance to closest TSS, *de novo* binding motif discovery, evolutionary conservation analysis and known motif mapping was performed using Cisgenome software packages [15] and custom built Perl scripts. For rank analysis, peaks from the final dataset were sorted by peak rank (calculated by Cisgenome package and dependent on read count as a measure of interaction strength) and divided into 10 bins. Bin 1 contains the 10% largest peaks bin 2 contains the range between 10 and 20%, etc as determined by the number of reads per peak.

### Microarray expression analysis of LS174T-L8 and Ls174T-pTER-β-catenin cells

Approximately 10^6^ Ls174T-L8 or Ls174T-pTER-β-catenin cells were grown in the presence or absence of doxycycline for 24 hours for Ls174T-L8 or 72 hours for Ls174T-pTER-β-catenin cells. Total RNA was extracted using TRIzol reagent (Invitrogen) according to the manufacturer's instructions. RNA concentration was determined using the NanoDrop ND-1000 and quality was determined using the RNA 6000 Nano assay on the Agilent 2100 Bioanalyzer (Agilent Technologies). For Affymetrix Microarray analysis, fragmentation of RNA, labeling, hybridization to HG-U133 Plus 2.0 microarrays, and scanning were carried out according to the manufacturer's protocol (Affymetrix Inc.). The expression data were normalized with the MAS5.0 algorithm within the GCOS program of Affymetrix. Target intensity was set to 100 (α1 = 0.04 and α2 = 0.06). Changes in the expression (logfolds and significance of change) for each of the comparisons were determined using the ‘Comparison Analysis’ from the GCOS program. All data were summarized using custom build Perl scripts. Genes were divided into three categories according to microarray expression data. Wnt upregulated genes - genes significantly down-regulated after suppressing Wnt dependent transcription by both ways in LS174T cells, in all 3 biological replicates (n = 647), ii) Wnt downregulated genes - genes significantly up-regulated after suppressing Wnt dependent transcription by two ways in all 3 biological replicates (n = 576) and iii) non-Wnt-regulated genes - genes consistently without significant expression change after suppressing Wnt-dependent transcription (n = 3936) (Table S1).

## Supporting Information

Table S1 **List of peak coordinates and ranks for TCF4, TBP, and H3K4me3 ChIP-seq experiments and Tcf4 up- and down-regulated genes.**
(XLS)

Figure S1 **Fifteen most overrepresented motifs found in Tcf4 binding regions.**
(DOC)

Figure S2 **Examples of de novo called motifs enriched in peaks.**
(DOC)

Figure S3 **Indirect immunoprecipitation of interacting regions.**
(DOC)

Figure S4 **Example of peaks called from Tcf4, TBP and H3K4me3 dataset.**
(DOC)
