# Supplementary material for: Efficient Double Fragmentation ChIP-seq Provides Nucleotide Resolution Protein-DNA Binding Profiles
Source: PLoS One. 2010 Nov 30;5(11):e15092. doi: 10.1371/journal.pone.0015092 (PMC2994895; doi:10.1371/journal.pone.0015092)
Supplement: Figure S1 — Fifteen most overrepresented motifs found in Tcf4 binding regions. (DOC) [file pone.0015092.s002.doc]

**Figure S1**


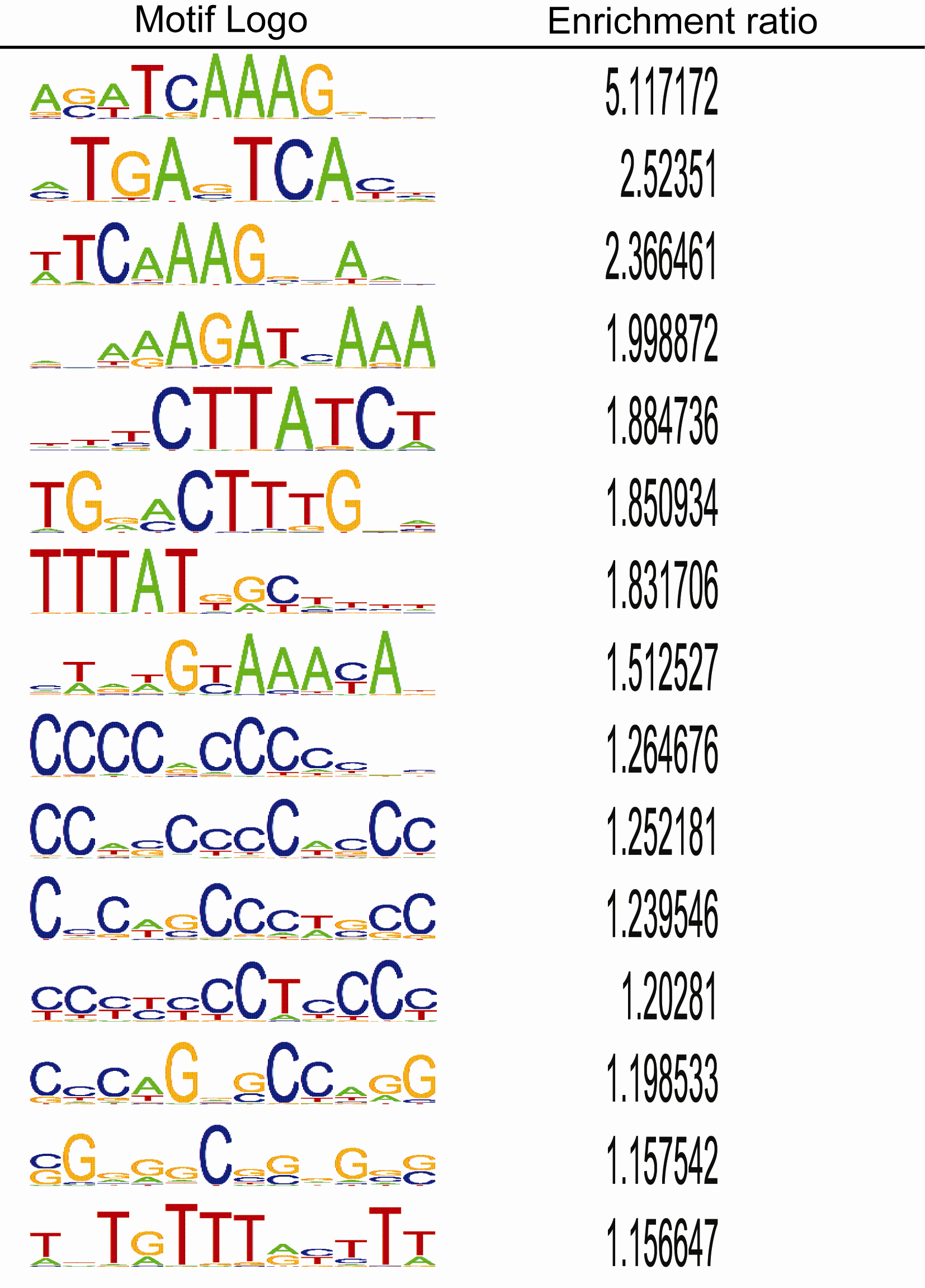


**Fig S1: Fifteen most overrepresented motifs found in Tcf4 binding regions.** Tcf4 binding motif shows highest enrichment in binding sites compared to the other motifs.
