# Supplementary material for: Efficient Double Fragmentation ChIP-seq Provides Nucleotide Resolution Protein-DNA Binding Profiles
Source: PLoS One. 2010 Nov 30;5(11):e15092. doi: 10.1371/journal.pone.0015092 (PMC2994895; doi:10.1371/journal.pone.0015092)
Supplement: Figure S2 — Examples of de novo called motifs enriched in peaks. (DOC) [file pone.0015092.s003.doc]

**Figure S2**


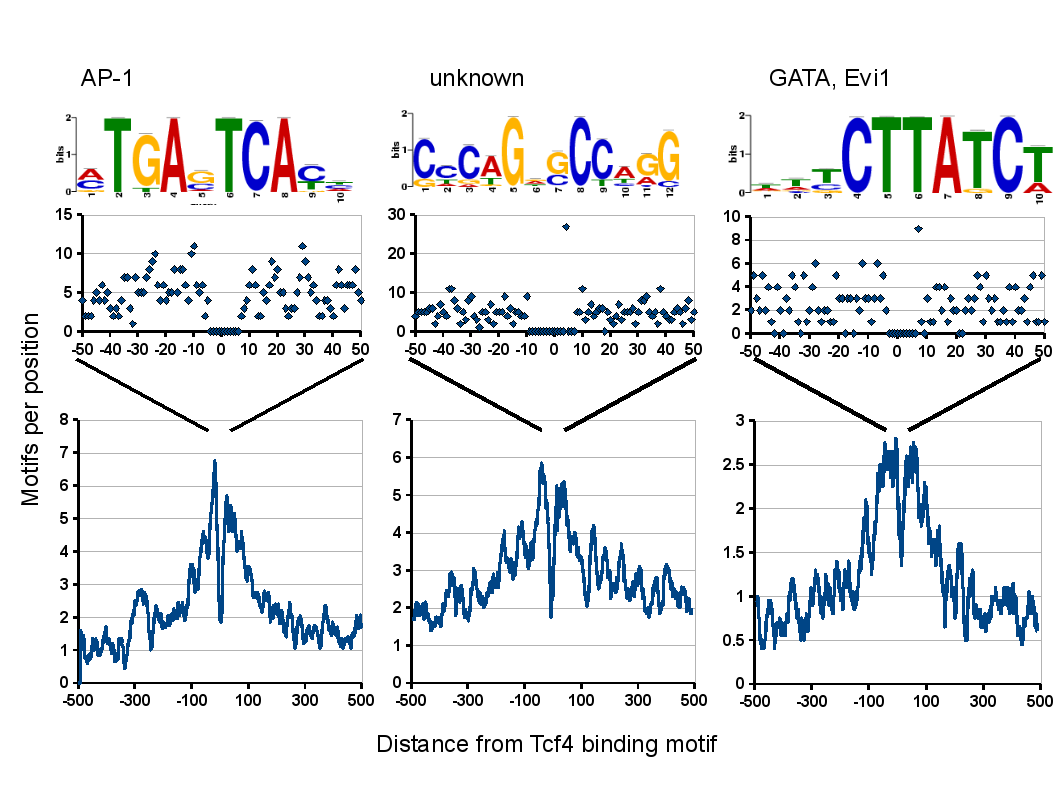


**Figure S2: Examples of *de novo* called motifs enriched in peaks.** Clustering of known protein binding motifs, such as AP-1 and GATA, close to the consensus Tcf4 binding motif supports the existence of possible functional interactions of these proteins with Tcf4. The middle motif is not associated with any known transcription factor and may represent the consensus binding site of a novel Tcf4-interacting partner.

**Identification of Tcf4 co-factors**

To identify DNA-binding proteins that could interact with Tcf4, we performed *de novo* identification of enriched sequence motifs in the immunoprecipitated regions and focused on the 15 most overrepresented motifs. The majority of the *de novo* called motifs showed clear enrichment close to known Tcf4 binding motifs in Tcf4 binding regions, often within 10 bp, suggesting a potential direct interaction with Tcf4 as a heterodimer. Alternatively, since Tcf4 harbors a HMG-box domain, which can increase flexibility of DNA [1,2] Tcf4 binding may primarily function to facilitate binding of other transcription factors in its neighborhood.

By comparing weighted matrices of *de novo* called motifs with binding motifs from the TRANSFAC database using the TOMTOM [3] web-based motif comparison tool, we identified binding motifs for several of the known Tcf4 interacting partners including SP1 [4], AP1 [5] and PPARγ [6]. In addition, we discovered novel binding motifs of proteins that were not associated with Tcf4 before and could thus represent binding sites for yet unidentified or uncharacterized Tcf4 interacting partners.

1. Love JJ, Li X, Case DA, Giese K, Grosschedl R, et al. (1995) Structural basis for DNA bending by the architectural transcription factor LEF-1. Nature 376: 791-795.

2. Ross ED, Hardwidge PR, Maher LJ, 3rd (2001) HMG proteins and DNA flexibility in transcription activation. Mol Cell Biol 21: 6598-6605.

3. Gupta S, Stamatoyannopoulos JA, Bailey TL, Noble WS (2007) Quantifying similarity between motifs. Genome Biol 8: R24.

4. Rossi A, Mukerjee R, Ferrante P, Khalili K, Amini S, et al. (2006) Human immunodeficiency virus type 1 Tat prevents dephosphorylation of Sp1 by TCF-4 in astrocytes. J Gen Virol 87: 1613-1623.

5. Nateri AS, Spencer-Dene B, Behrens A (2005) Interaction of phosphorylated c-Jun with TCF4 regulates intestinal cancer development. Nature 437: 281-285.

6. Jansson EA, Are A, Greicius G, Kuo IC, Kelly D, et al. (2005) The Wnt/beta-catenin signaling pathway targets PPARgamma activity in colon cancer cells. Proc Natl Acad Sci U S A 102: 1460-1465.
