# Supplementary material for: Efficient Double Fragmentation ChIP-seq Provides Nucleotide Resolution Protein-DNA Binding Profiles
Source: PLoS One. 2010 Nov 30;5(11):e15092. doi: 10.1371/journal.pone.0015092 (PMC2994895; doi:10.1371/journal.pone.0015092)
Supplement: Figure S3 — Indirect immunoprecipitation of interacting regions. (DOC) [file pone.0015092.s004.doc]

**Figure S3**


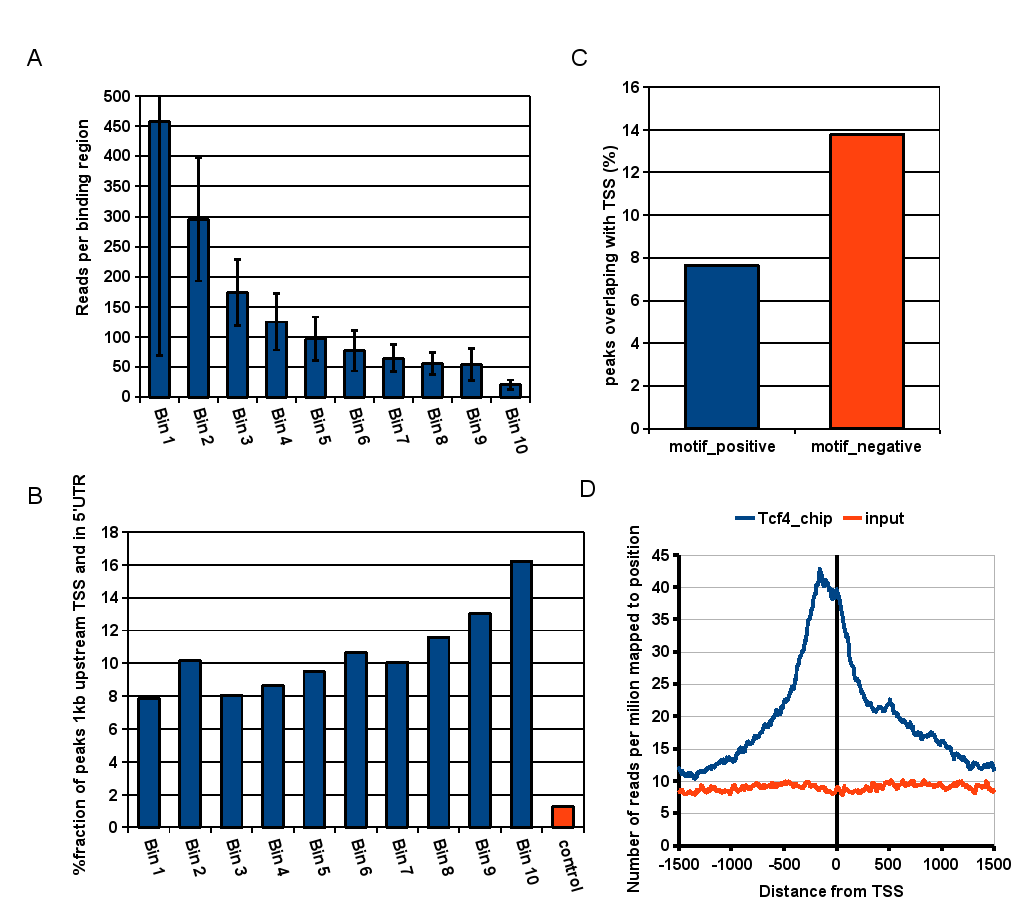


**Figure S3: Indirect immunoprecipitation of interacting regions.** A) The average number of sequencing reads mapped to the binding region with respect to protein-DNA interaction strength. Approximately 50% of the sequencing reads that map to binding regions map to 10% of the strongest ones (determined by the number of reads mapping to the binding region). B) Distribution of binding regions in relation to the closest protein-coding gene with respect to protein-DNA interaction strength. Weaker binding regions tend to overlap more often with transcription start sites (TSS). C) Percentage of binding regions mapped to TSS with respect to the presence of the Tcf4 consensus binding motif. D) Enrichment pattern of sequencing tags around TSS compared to the pattern of control sequencing tags from input DNA.

**Identification of chromatin interactions**

In contrast to ChIP-chip, ChIP-seq has a very high dynamic range and allows for semi-quantitative estimation of DNA-protein interaction strength (as a function of the number of sequencing reads which mapped to the binding region) [1]. Furthermore, lowly represented weak binding regions are more easily detected as the ChIP-seq output has a ‘digital’ nature (read counts) without the need for complicated background corrections. Extensive characterization of the distribution of Tcf4 binding regions revealed an unequal size distribution among peaks. The top 10 % of the strongest binding regions (as determined by the number of reads contributing to the peak) contain about ~50 % of all reads that map to peaks, suggesting that the majority of Tcf4 is bound to only a limited number of binding sites. The biological relevance of the remaining weaker binding peaks is difficult to interpret as i) the interaction itself could be weaker, potentially resulting from stabilized binding to random or suboptimal sites, ii) the interaction may only be present in a limited number of cells from the whole population, iii) Tcf4 can be less accessible by antibody on those regions or iv) they could represent biologically irrelevant noise. However, these weak binding sites could also be biologically relevant as they may represent other types of interactions, such as co-immunoprecipitated chromatin that interacts indirectly via DNA-looping with Tcf4-containing protein-DNA [2]. To address this, we sorted the peaks according to the number of mapped sequencing reads and divided them into 10 bins. Sites covered by fewer reads (in the higher number bins) tend to occur more often close to transcription start site (TSS) positions than sites with more reads. As Tcf4 is considered to be primarily an enhancer-binding factor, the strong peaks are likely to reflect the real Tcf4 binding sites. Since indirect immunoprecipitation is expected to be less efficient, the frequency of reads representing such interactions can be expected to be relatively low. The observed increased number of weaker peaks overlapping with TSS supports the hypothesis that a substantial part of the identified Tcf4 binding regions actually originate from indirect immunoprecipitation of proteins interacting with Tcf4, similar to what was shown previously for the estrogen receptor [2]. In support of this, a large proportion of weak binding regions (44.5%) was found to be devoid of consensus Tcf4 binding motifs. In general, Tcf4 motif-negative regions do overlap more often with TSS regions, compared to peaks with a clear Tcf4 binding motif. Other evidence that supports indirect immunoprecipitation of promoters of genes that are regulated by Tcf4 comes from the typical asymmetric overall substructure of these peaks. Interestingly, this pattern is remarkably similar to patterns observed for RNA polymerase II ChIP-seq in promoter regions of active genes [3,4], strongly suggesting functional physical interactions between Tcf4 enhancer complexes and the transcription machinery.

1. Jothi R, Cuddapah S, Barski A, Cui K, Zhao K (2008) Genome-wide identification of in vivo protein-DNA binding sites from ChIP-Seq data. Nucleic Acids Res 36: 5221-5231.

2. Carroll JS, Meyer CA, Song J, Li W, Geistlinger TR, et al. (2006) Genome-wide analysis of estrogen receptor binding sites. Nat Genet 38: 1289-1297.

3. Yang MQ, Elnitski LL (2008) Diversity of core promoter elements comprising human bidirectional promoters. BMC Genomics 9 Suppl 2: S3.

4. Sultan M, Schulz MH, Richard H, Magen A, Klingenhoff A, et al. (2008) A global view of gene activity and alternative splicing by deep sequencing of the human transcriptome. Science 321: 956-960.
