# Supplementary material for: Efficient Double Fragmentation ChIP-seq Provides Nucleotide Resolution Protein-DNA Binding Profiles
Source: PLoS One. 2010 Nov 30;5(11):e15092. doi: 10.1371/journal.pone.0015092 (PMC2994895; doi:10.1371/journal.pone.0015092)
Supplement: Figure S4 — Example of peaks called from Tcf4, TBP and H3K4me3 dataset. (DOC) [file pone.0015092.s005.doc]

Figure S4


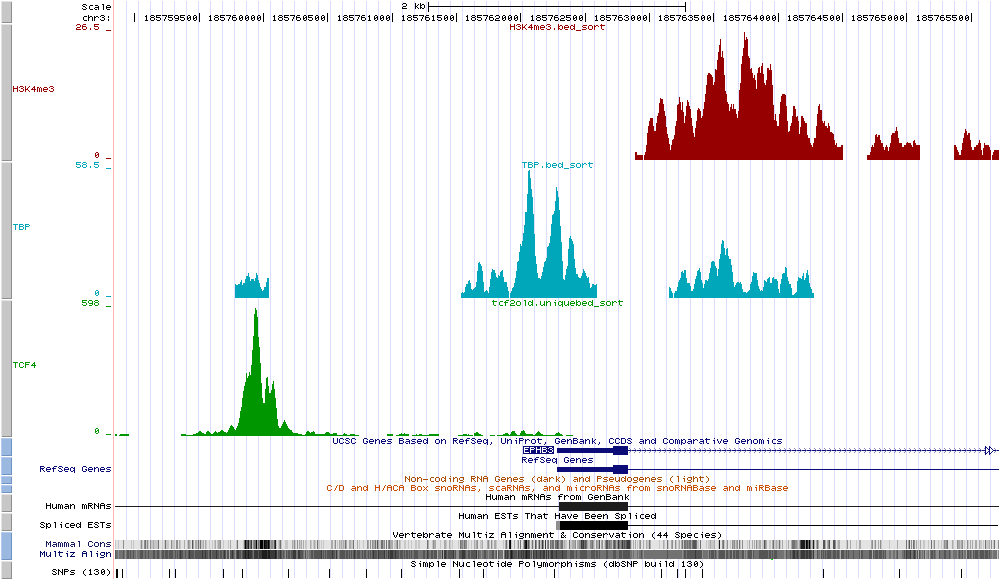


**Figure S4: Example of peaks called from Tcf4, TBP and H3K4me3 dataset.** Small but significant TBP peak is present on position of Tcf4 peak located upstream of expected transcription start site of EPHB3 gene. Additionally, small Tcf4 peak is present directly at transcription start site of EPHB3 gene on position where main TBP peak is present. This could suggest that these small peaks originate in indirect immunoprecipitation of TBP bound region interacting with Tcf4 and vice versa.
